# Biology, genetics, and ecology of the cosmopolitan ectomycorrhizal ascomycete *Cenococcum geophilum*

**DOI:** 10.3389/fmicb.2025.1502977

**Published:** 2025-01-23

**Authors:** Huayong Wang, Annegret Kohler, Francis M. Martin

**Affiliations:** ^1^Université de Lorraine, INRAE, UMR Interactions Arbre/Micro-organismes, Centre INRAE Grand-Est Nancy, Champenoux, France; ^2^The National Key Laboratory of Ecological Security and Sustainable Development in the Arid Region, Northwest Institute of Eco-Environment and Resources, Chinese Academy of Sciences, Lanzhou, China

**Keywords:** drought, environmental stress, forest, genomics, mycorrhizal symbiosis, population genetics

## Abstract

The ascomycete *Cenococcum geophilum* is a cosmopolitan and ecologically significant ectomycorrhizal (ECM) fungus that forms symbiotic associations with diverse host plants worldwide. As the only known ECM species within the large class Dothideomycetes, *C. geophilum* exhibits several characteristics that distinguish it from other ECM fungi. This fungus significantly contributes to ecosystem stability and development as an early colonizer of primary forest succession. The capacity of this symbiont to rapidly colonize disturbed or newly formed environments promotes the development of conditions that support the growth of other plant species, thus playing a crucial role in the ecological progression and restoration of ecosystems. Several *C. geophilum* isolates are known to enhance the drought resistance of host plants, a trait that is becoming increasingly important in the context of climate change and frequent drought events. In this review, we examined genetic studies that have assessed the phylogenetic structure of *C. geophilum* populations and identified the genes associated with adaptation to environmental stress and symbiosis. The high genetic diversity of *C. geophilum* is particularly noteworthy, considering its putative asexual reproductive mode. Population genomic analyses have suggested that *C. geophilum* is not a single species but rather a species complex comprising multiple cryptic lineages. This genetic variability may contribute to its adaptability and extensive distribution across habitats from circumpolar to tropical biomes. These lineages exhibit potential host preferences, suggesting a degree of specialization within the complex. The nuclear genome of *C. geophilum* has been sequenced, providing valuable insights into the symbiont genetic traits. Notably, this genome encodes a large set of repeated sequences and effector-like small secreted proteins. Transcriptomics has been used to identify candidate genes related to symbiosis and adaptation to environmental stress. Additionally, we briefly discuss how *C. geophilum* offers potential for sustainable forestry practices by improving resilience to stress.

## 1 Introduction

A majority of land plants establish symbiotic relationships with mycorrhizal fungi, which play a critical role in terrestrial ecosystems by regulating nutrient and carbon cycles, influencing soil structure, and contributing to ecosystem multifunctionality (Martin and Van Der Heijden, 2024). Approximately 80% of plant N and P are provided by these mutualistic fungi, and the majority of plant species depend on them for growth and survival. An estimated 20,000 fungal species, primarily belonging to the phyla Basidiomycota and Ascomycota, establish ectomycorrhizal (ECM) associations with approximately 6,000 plant species, mostly trees and shrubs (Van Der Heijden et al., 2015). ECM fungi are present in a diverse range of terrestrial ecosystems and are responsible for colonizing 60% of the trees in temperate and boreal forest ecosystems (Baldrian et al., 2023). These tree species, belonging to the Pinaceae, Fagaceae, Betulaceae, Nothofagaceae, Myrtaceae, or Dipterocarpaceae families, play crucial ecological and economic roles in both the northern and southern hemispheres.

During symbiosis development, ECM fungi differentiate the hyphal mantle, ensheathing the rootlets and an intraradical mycelial network, the so-called Hartig net, which penetrates host roots. In numerous ECM associations, an extraradical mycelium permeating the soil environment extends from ECM roots. Mycelial networks facilitate the acquisition of water and nutrients by plants and enhance their resistance to environmental stressors. ECM symbionts secrete extracellular enzymes that degrade soil organic matter (SOM) to facilitate nitrogen acquisition in their hosts (Nicolas et al., 2019). ECM fungi from different independently evolved lineages exhibit varying capacities to degrade SOM and transfer N to their hosts (Nicolas et al., 2019). In boreal and temperate forests, ECM fungi provide 70% of N flux to their hosts (Smith and Read, 2010). Consequently, ECM plays a crucial role in C and N cycles in forest soils. In exchange for soil minerals, 10–20% of photoassimilates are allocated to fungal partners by the host plant. Plant communities allocate 9.07 Gt of atmospheric CO_2_ per year to their mycorrhizal symbionts (Hawkins et al., 2023).

**Figure 1:**
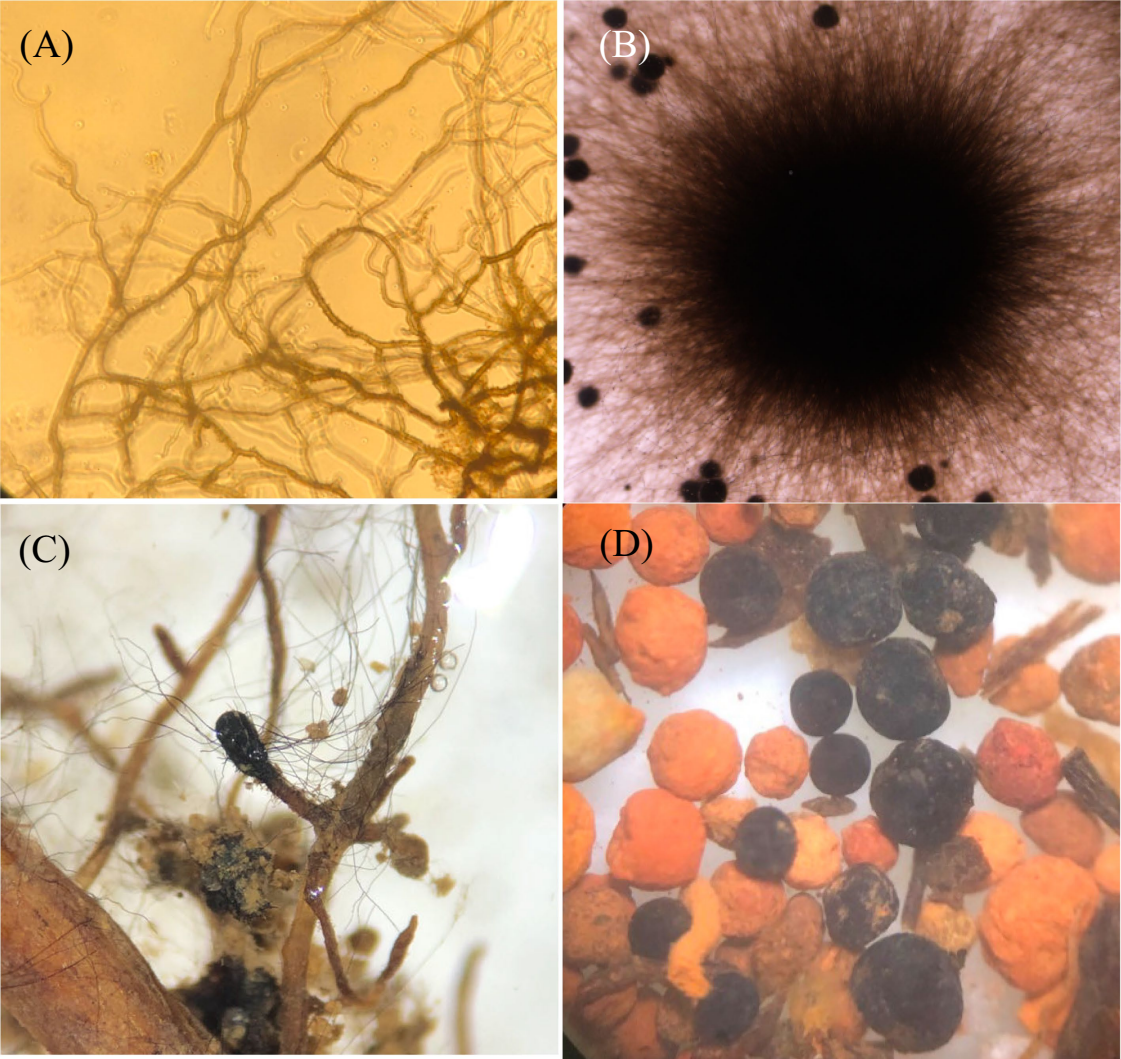
The ascomycete *Cenococcum geophilum*: **(A)** melanized mycelial hyphae, **(B)** vegetative colony of free-living mycelium, **(C)** an ectomycorrhizal root tip of the *Fagus sylvatica-Cenocccum geophilum* association with extraradical hyphae, and **(D)** melanized sclerotia.

The ascomycetous fungus *Cenococcum geophilum*, previously known as *C. graniforme*, is a cosmopolitan ECM fungus and one of the most prevalent mutualistic species found in soil fungal communities worldwide (LoBuglio, 1999; Figure 1). It forms mycorrhizal associations with over 200 trees, shrubs, and herbaceous species in boreal, temperate, and subtropical forests as well as in savannas and alpine meadows. As the only known ECM member of the class Dothideomycetes, *C. geophilum* exhibits several distinctive characteristics that distinguish it from other ECM fungi (LoBuglio, 1999; Obase et al., 2017). As an early colonizer of primary forest succession, *C. geophilum* contributes significantly to ecosystem stability and development. It is particularly important in nutrient cycling because it facilitates the transfer of nutrients, especially nitrogen (N) and phosphorus (P), from the soil to its host plants (LoBuglio, 1999). Additionally, *C. geophilum* enhances the drought resistance of its host plants (Coleman et al., 1989), a trait that is becoming increasingly important in the context of a warming world with an increased occurrence of drought events (Zheng et al., 2023). *C. geophilum* is therefore a compelling model system for research on fungal ecology, evolution, and mycorrhizal symbiosis.

The biological and ecological attributes of *C. geophilum* have been documented extensively (LoBuglio, 1999; Obase et al., 2017). Recently, genomics has emerged as a crucial tool for investigating the biology, evolution, and ecology of mutualistic symbionts including *C. geophilum* (Kohler et al., 2015; Peter et al., 2016; Miyauchi et al., 2020; Lebreton et al., 2021). This approach not only provides essential mechanistic insights but also identifies key genetic traits, such as adaptation to drought stress (Li et al., 2023; Zhang et al., 2024), which can be prioritized to select strains for the application of this mycorrhizal symbiont in forestry. This review provides a brief account of the biological and ecological attributes of *C. geophilum*, followed by a discussion of recent studies that have demonstrated the impact of genomics and related techniques (i.e., DNA metabarcoding, population genomics, and transcriptomics) on our understanding of this enigmatic mycorrhizal fungus. Additionally, it briefly explores the potential applications of *C. geophilum* in sustainable forestry and ecosystem restoration, highlighting the significance of understanding the functional traits and ecological roles of these ECM fungi in adapting to environmental changes. By consolidating the latest research findings, this review aims to identify knowledge gaps and suggest future research directions for this ubiquitous symbiont to address the global challenges in forestry and environmental sustainability.

## 2 Morphological features and life cycle

The black fungus *C. geophilum* is distinguished by its septate dematiaceous hyphae, which contain high concentrations of melanin in their cell walls (Figure 1; Fernandez and Koide, 2013). This pigmentation enables mycelia to endure various environmental challenges including UV exposure, dehydration, high temperatures, enzymatic breakdown, antimicrobial agents, and heavy metal exposure (Pal et al., 2013). The resilience of *C. geophilum* enables it to thrive in challenging environments for several years, where other mycorrhizal fungi may find it difficult to survive (McCormack et al., 2017). Its hyphae show various shapes according to the growth medium and the age of the mycelial colony (Trappe, 1962). Chlamydospore-like structures have been observed in both solid and liquid media (Massicotte et al., 1992). These chlamydospore-like structures are always intercalary and rarely terminal in the mycelia (Mikola, 1948). This structure also exists in the taxonomically related species *Glonium* spp. (Amano, 1983) and *Pseudocenococcum floridanum* (Obase et al., 2016).

*Cenococcum geophilum* can differentiate sclerotium (Figure 1), which is a compact mass of hardened fungal mycelium containing nutrient reserves, including carbohydrates and lipids. The sclerotia constitute an underestimated source of polysaccharides in forest soils, accounting for 3.6% of the total carbohydrates in subalpine forest soils (Murayama and Sugiura, 2021). These melanized sclerotia resist decomposition by soil microorganisms (Fernandez and Koide, 2014) and remain viable for up to 40 years under extreme environmental conditions (Nyamsanjaa et al., 2022). They host specific fungal and bacterial communities (Obase et al., 2014; Narisawa et al., 2021).

Although molecular evidence, such as recombination and diploidy (see below), suggests the presence of unknown sexual stages in the life cycle of *C. geophilum*, no sexual structures have been observed under laboratory or field conditions (Bourne et al., 2014).

The only ECM fossil related to *C. geophilum* is *Eomelanomyces cenococcoides* gen. Spec. nov., discovered in a 52-million-year-old amber specimen from a lignite mine in Gujarat State, India (Beimforde et al., 2011). This amber was produced by representatives of Dipterocarpaceae trees in the early tropical broadleaf forests. The fossil is similar to the extant *Cenococcum*; however, it is distinguished by high variability in the branching of ECM rootlets and by the regular formation of microsclerotia and chlamydospore-like structures (Beimforde et al., 2011).

## 3 Ecologically important ectomycorrhizal symbiont

**Figure 2:**
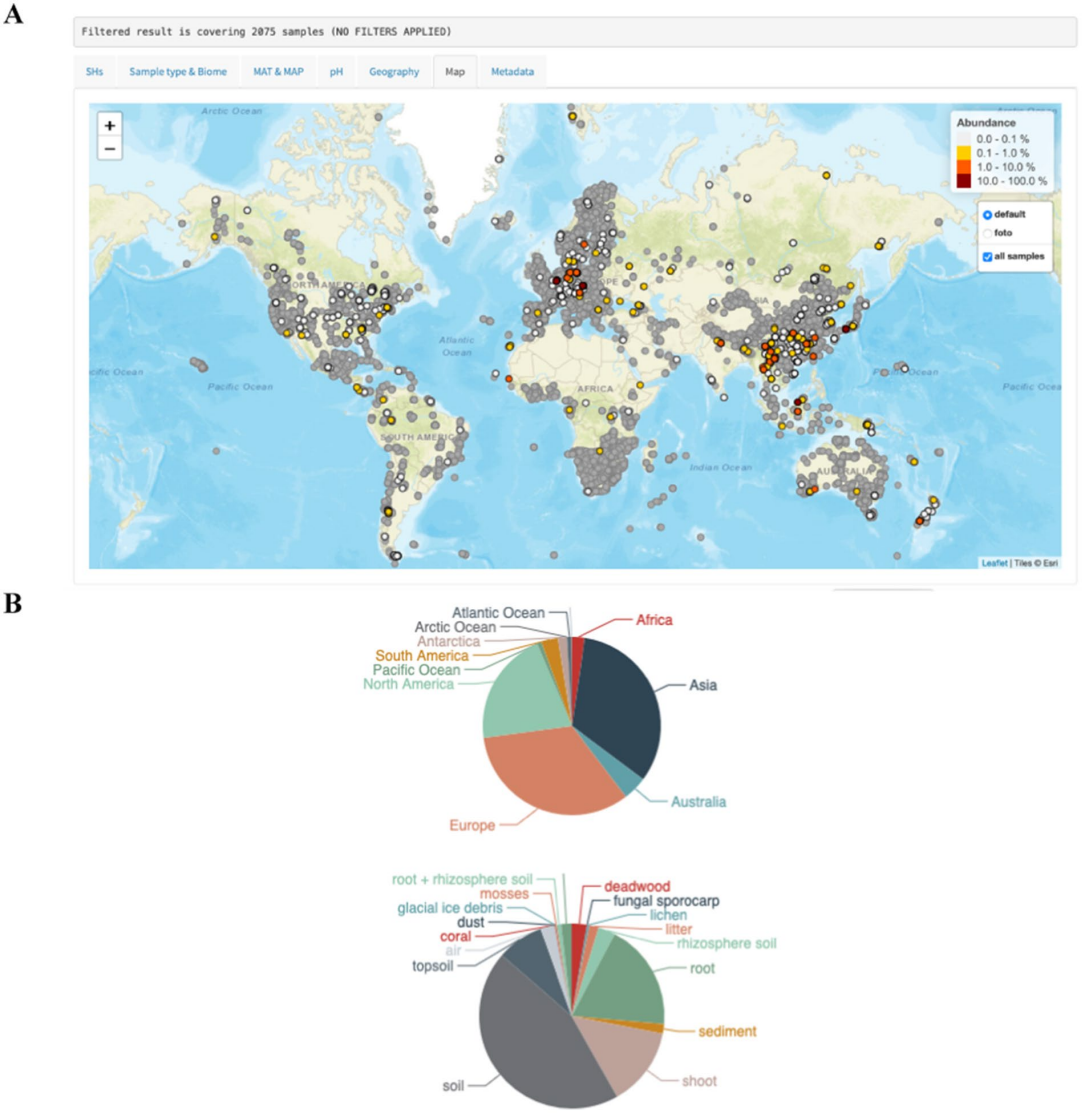
Assessment of the worldwide abundance and distribution of *Cenococcum geophilum* **(A)** and its distribution (% per site) among the biomes and continents **(B)** using the GlobalFungi database (https://globalfungi.com) (Větrovský et al., 2020).

The identification of *C. geophilum* relies on a combination of morphological and molecular techniques, as it shares soil habitats and many physical characteristics with dark septate root endophyte (DSE) fungi such as *Piceirhiza bicolorata* and *Cadophora finlandia* (Rosling et al., 2003). DNA metabarcoding surveys have shown that this ECM fungus is a major component of the soil fungal communities in most of the forest ecosystems (Figure 2). It is considered a keystone species essential for maintaining the microbial network structure and stability (Zhu et al., 2024). As an early colonizer in primary successions, *C. geophilum* significantly contributes to ecosystem stability and development (LoBuglio, 1999). Its rapid establishment in disturbed or newly formed habitats creates favorable conditions for other plant species, thus playing a vital role in ecological succession and ecosystem recovery. Additionally, *C. geophilum* can collaborate with other bacteria to establish ECM associations under varying climatic conditions. Reis et al. (2021) examined beneficial symbiotic microorganisms, including ECM fungi and mycorrhiza helper bacteria in cork oak (*Quercus suber* L.) forests. *C. geophilum* and *Bacillus* sp. were among the most prevalent interacting microbes. Furthermore, Kataoka et al. (2009) reported that *B. subtilis* can enhance *C. geophilum* growth during symbiosis establishment. This mutual support benefits all three partners and could play a crucial role in forest resilience to future climate change.

## 4 The pan-global ectomycorrhizal symbiont

*Cenococcum geophilum* is a cosmopolitan ECM fungus and one of the most prevalent species found in soil fungal communities worldwide (Figure 2). It forms ECM or ectendomycorrhizal associations with over 200 trees, shrubs, and herbaceous species in boreal, temperate, and subtropical forests as well as in savannas. In alpine and circumpolar biomes, *C. geophilum* establishes ectendomycorrhizas or ECM with herbaceous plants such as sedges or shrubs (Obase et al., 2017). Its abundance in tropical ecosystems is relatively low (Tedersoo et al., 2010; Bakray et al., 2024), although high root colonization and genetic diversity have been reported in the dry deciduous forests of Thailand (Phosri et al., 2012). However, *C. geophilum* ECM is seldom found in African or South American tropical forests (Becerra and Zak, 2011; Bâ et al., 2012). It has also been found at the edges of deserts (Massicotte et al., 1992) and in sandy forests of *Picea mongolica* (Bao, 2005).

Zheng et al. (2023) employed the MaxEnt model (Phillips et al., 2017) to analyze the historical shifts in geographical distribution patterns of *C. geophilum* since the Last Glacial Maximum and forecast its future spread under changing climatic conditions. They showed that this geographical distribution is closely associated with climatic factors, particularly temperature and precipitation. Temperature has greater relative importance than precipitation. This is also true for majority of the ECM fungi (Bennett and Classen, 2020). *C. geophilum* occupied a significantly smaller area during the Last Glacial Maximum and mid-Holocene, primarily concentrated in China’s Qinling–Huaihe Line region and eastern Peninsular Malaysia. As global temperatures continue to rise, the model predicts a northward shift toward a suitable habitat for *C. geophilum*, resulting in an anticipated increase in suitable areas from 9 to 21%.

*Cenococcum geophilum* ECM rootlets are abundant in the top 0–5 cm soil layer (Rosling et al., 2003; Genney et al., 2006; Scattolin et al., 2008) but can also be found in much deeper soil layers, such as the mineral layer at a depth of 20 cm (Genney et al., 2006). *C. geophilum* is a pioneer species because of its propensity to partner with pioneer host trees such as *Salix* spp., which colonize newly exposed glacier moraines (Trappe, 1988). Moreover, the symbiont is recognized as a “multi-stage” fungus in secondary forest successions, indicating that it forms ECM associations with both seedlings and adult host plants (Visser, 1995; Danielson, 1991). In the volcanic desert of Mount Fuji, *C. geophilum* is present in both the early and later stages of vegetation development, colonizing young and old pioneer shrubs, such as *Salix reinii*, and herbaceous species, such as *Polygonum cuspidatum* (Nara, 2006). Interestingly, the symbiont has been found in old growth forests (Peter, 2003), although it is known to primarily colonize young trees in alpine regions near treelines (Hasselquist et al., 2005). In particular, *C. geophilum* is associated with seedlings and juvenile trees of *Picea engelmannii* and *Abies lasiocarpa*, with colonization rates 20 times greater for juveniles than for seedlings (Hasselquist et al., 2005). These findings suggest that this fungus plays an important role in the early stages of forest succession. However, *C. geophilum* has not been replaced by late-stage ECM species in older forest stands. The high prevalence of *C. geophilum* in mature alpine forest ecosystems, which are known for their cold climate, slow litter breakdown, and organic matter buildup in the soil, is believed to be a consequence of the substantial presence of sclerotia (approximately 3,600 kg ha^−1^) and synchronization of rootlet growth bursts with sclerotia germination in autumn (Vogt et al., 1982). Furthermore, *C. geophilum* is among the most frequent ECM symbionts following a short fire return interval (Buscardo et al., 2010).

Mineral weathering by *C. geophilum* can release potassium from potassium aluminosilicate minerals, such as feldspar, nepheline, biotite, muscovite, and illite (Xue et al., 2019). The symbiotic fungus can also break down the mycorrhizal necromass (Fernandez and Koide, 2014; Gray and Kernaghan, 2020), with the initial N and melanin levels strongly influencing the early decay rates and determining the remaining mass after several years.

## 5 Tolerance to N deposition, salt stress, and heavy metals

N addition reduced the prevalence of *C. geophilum* in the fungal communities of the humus and fine roots. Forsmark et al. (2024) analyzed the organic layer beneath undisturbed litter in a Norway spruce (*Picea abies*) forest in northern Sweden after two decades of annual N application at low (12.5 kg N ha^−1^ yr.^−1^) and high (50 kg N ha^−1^ y^r − 1^) levels. N supplementation decreased *C. geophilum* abundance, suggesting that decomposition linked to organic N acquisition was suppressed when inorganic forms of N were readily accessible. These community changes were associated with a decreased activity of Mn-peroxidase and peptidase and an increase in the activity of C-acquiring enzymes.

Wen et al. (2022) studied the influence of *C. geophilum* inoculation on the growth and nutrient uptake of *Pinus thunbergii* seedlings under salt stress. Their results indicated that mycorrhizal inoculation significantly increased seedling biomass, chlorophyll, and nutrient elements (such as P, N, and K) in shoots and maintained a low Na/K ratio in roots under salt stress, suggesting that inoculation with *C. geophilum* could assist the host in overcoming salt stress. Geographical isolates of *C. geophilum* have shown patterns of local adaptation to serpentine soils, with Ni concentrations having a significant effect on fitness-related traits (Gonçalves et al., 2009; Bazzicalupo et al., 2020).

## 6 Adaptation to water-stressed environments

*C. geophilum* exhibits drought tolerance and is prevalent in water-stressed environments (Pigott, 1982; McCormack et al., 2017). Several surveys of soil fungal communities have demonstrated that the proportions of *C. geophilum* ECM and extramatrical mycelia increase under water stress conditions and are often higher during summer in natural settings (Pigott, 1982; Querejeta et al., 2009). This tolerance has been verified through *in vitro* mycelial culture experiments using osmotically adjusted media (Mexal and Reid, 1973; Coleman et al., 1989), cell damage tests following desiccation (Di Pietro et al., 2007), and respiration measurements under water stress (Jany et al., 2003). The level of tolerance varies among geographical isolates (Coleman et al., 1989; Jany et al., 2003). However, the physiological mechanisms responsible for this symbiont’s success under water stress remain largely unknown. Multiple factors likely contribute to this trait, such as the accumulation of compatible osmolytes (e.g., polyols), heat shock proteins, hydrophobic proteins, and melanin in the cell walls. It has also been suggested that drought resistance in *C. geophilum* may be associated with the increased expression of aquaporin water channels (see below, Peter et al., 2016). Although *C. geophilum* is widely recognized as a drought-tolerant symbiont, this contention has recently been debated. A study utilizing *Pinus* seedlings colonized by *C. geophilum* and subjected to water shortages showed that the drought resistance of mycorrhizal plantlets was not directly correlated with that of *C. geophilum* isolates cultivated in liquid medium (Zhang et al., 2024). Xie et al. (2024) used inoculated *Quercus mongolica* and *Tilia amurensis* to investigate the responses of ECM fungal communities and their exploration types under drought conditions in a pot system. The relative abundance of *C. geophilum* in both hosts decreased. Nickel et al. (2018) examined ECM fungal community diversity changes in European beech and Norway spruce forests under drought conditions by utilizing retractable roofs to exclude rain for 3 years. The results indicated that the abundance of *C. geophilum* decreased irrespective of the depth, year, or host.

## 7 Heat and cold stresses

Laboratory experiments demonstrated that the growth inhibition of several *C. geophilum* isolates occurred at a temperature of 26°C (Yan et al., 2022); however, this species is capable of forming mycorrhizal associations following exposure to heat stress at approximately 70°C for a brief period or at 5°C above ambient temperature. Nevertheless, combined stress, including drought and heat stress, at a temperature of 5°C above ambient temperature and 50% precipitation can be lethal to *C. geophilum* (Kipfer et al., 2010; Gehring et al., 2020). Herzog et al. (2013) reported that increased temperature and water shortage can differentially affect the relative ECM abundance and exoenzyme activities of *C. geophilum* associated with various oak species, specifically *Q. robur*, *Q. petraea*, and *Q. pubescens*.

Furthermore, because of their prevalence as symbionts in arctic and alpine ecosystems, *C. geophilum* mycelia and ECM are likely to exhibit high tolerance to cold stress. Corbery and Le Tacon (1997) demonstrated that *C. geophilum* mycelium remained viable even when exposed to a freezing temperature of −80°C for a brief period, exhibiting greater resistance to cold than other ECM fungi. Additionally, studies have indicated that this fungus thrives at temperatures below 1°C (Vogt et al., 1982). This cold stress resistance may be attributed to its high mannitol synthesis rate (Martin et al., 1985) because mannitol is known to shield fungi from severe cryoenvironments (Weinstein et al., 1997).

## 8 Host preferences

*C. geophilum* is recognized as a mycorrhizal generalist species. This symbiont can form ecto-or ectendomycorrhizal associations with a broad host range. Based on the morphology and anatomy of mycorrhizal roots sampled in natural settings, three groups of host plants were identified (Trappe, 1962; LoBuglio, 1999): In Group 1, the hosts include members of the Salicaceae and Betulaceae families (excluding *Corylus* spp.), as well as ectotrophic genera within the Rosaceae family. The ECM root tips are typically monopodial or occasionally branched, with the mantle covering only the root tips. The Hartig net in these hosts never extends deeper than the third layer of the cortical cells, and intracellular penetration is sparse and limited to occasional cells. In Group 2, *C. geophilum* associates with *Pinus* species. The ECM root tips are monopodial, dichotomous, or occasionally irregularly branched. The mantles typically cover all short roots and have thicknesses ranging between 8 and 60 μm. The Hartig net extends inward to the innermost layer of cortical cells, and the cortex experiences strong intracellular infection. The hosts in Group 3 predominantly comprise Fagaceae, including *Corylus*, and Pinaceae, with the exception of *Pinus* spp. The root tips of these associations exhibit a range of morphologies, including monopodial, racemose, irregularly branched, long, or short structures. The mantle typically covers a significant portion or all of the short roots, and its thickness ranges from to 8 to 60 μm. The Hartig net extends to the innermost layer of the cortical cells, and intracellular infection is prevalent throughout the cortex.

Additionally, this groups includes many shrubs and herbaceous plants such as *Pedicularis capitata* (Kohn and Stasovski, 1990), *Cistus* spp. (Massicotte et al., 2010), *Bistorta vivipara* (Massicotte et al., 1998), *Carex myosuroides* (Massicotte et al., 1998), and *Rhododendron* spp. (Largent et al., 1980; Vohník et al., 2007). Unusual for an ECM symbiont, *C. geophilum* can also establish ectendomycorrhizal associations with shrubs and herbaceous plants, sharing mycelial networks with woody plants such as oak and *Helianthemum bicknellii* (Dickie and Reich, 2005), or the *Dryas octopetala*–*Bistorta vivipara*–*Salix herbacea* association (Mühlmann and Peintner, 2008). Symbiosis with herbaceous plants appears to enhance the colonization of woody plants (Dickie and Reich, 2005; Hoeksema et al., 2018). Although there is no evidence of nutrient transfer between herbaceous and woody plants sharing common mycorrhizal networks (CMNs) with *C. geophilum*, this structure could possibly act as a physical link between roots of herbaceous and woody plants, thereby enhancing *C. geophilum* colonization in sharing plants. The CMNs may also alter the bacterial communities of the hyphosphere (Vik et al., 2013).

Variations in colonization rates and/or host preferences can be attributed to genetic factors in both partners, as well as environmental factors such as soil organic matter content, total N, and available P (Wurentaoges, 2012). Zhu et al. (2024) reported that leaf photosynthesis and root morphological traits drive the topological structure of plant–fungus association networks involving *Cenococcum* species. Abundant plants may play a key role as reservoirs of symbiotic fungal diversity and thus contribute to the maintenance of ecosystem functions.

## 9 Population structure

As previously mentioned, *C. geophilum* is widespread and has historically posed challenges in terms of physiological and phylogenetic classification. Collections of *C. geophilum* isolates, both locally and globally, have shown remarkable genetic diversity. Genetic studies on *C. geophilum* have revealed a complex population structure, even at the soil core sample level, with evidence of both local adaptation and limited gene flow between populations (Jany et al., 2002; Douhan and Rizzo, 2005; Matsuda et al., 2015; Obase et al., 2016; Obase et al., 2017; Vélez et al., 2021). They uncovered the presence of multiple hidden clades and distinct phylogenetic groups within *C. geophilum*, supporting the widely held view that this species represents a highly diverse assemblage of ectomycorrhizal fungi at regional and global levels. The structure of symbiont populations is influenced by several factors, including geographic distance, environmental gradients, and host–plant associations. They are often structured according to environmental conditions such as soil type, moisture level, and temperature. For example, populations from dry nutrient-poor soils tend to be genetically distinct from those in more fertile environments, suggesting a local adaptation to specific ecological niches (Douhan and Rizzo, 2005; Lian et al., 2006). A subtle geographic structure with long-distance disjunction suggests complex alternation of sexual and asexual reproduction over space and time (Obase et al., 2016; Obase et al., 2017). However, gene flow between populations can occur through sclerotia dispersal, leading to a combination of local adaptation and genetic exchange.

**Figure 3:**
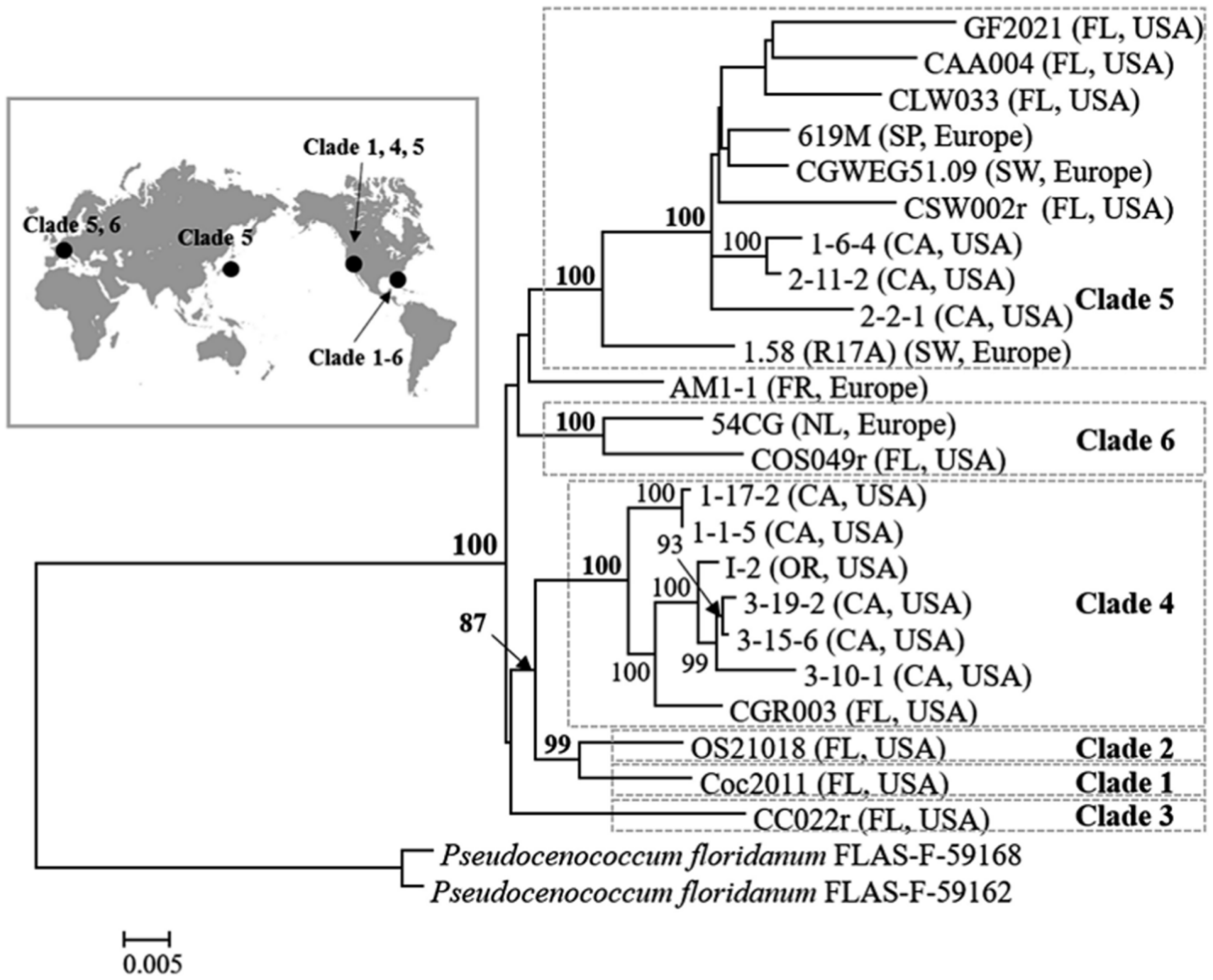
Phylogenetic tree of *Cenococcum geophilum*: major clades and their worldwide distribution (CA, California; FL, Florida; FR, France; NL, Netherlands; OR, Oregon; SP, Spain, SW, Switzerland). A maximum-likelihood phylogenetic tree was constructed using seven concatenated loci (ITS, SSU, LSU, TEF, RPB1, RPB2, and GAPDH). Isolates of *Pseudocenococcum floridanum* were used as outgroups (Obase et al., 2017).

The presence of cryptic species within *C. geophilum* has also been confirmed, with distinct genetic lineages corresponding to different ecological and geographical regions (Obase et al., 2017; Vélez et al., 2021). Obase et al. (2017) resolved seven clades with high bootstrap support among the isolates of *Cenococcum* derived from different geographical regions across the world using both single-and multi-locus and maximum likelihood (ML) analyses (Figure 3). All *Cenococcum* clades clustered together with high bootstrap support, whereas *Pseudocenococcum floridanum* isolates were resolved as a separate group. More recently, Vélez et al. (2021) examined a set of 200+ *C. geophilum* isolates obtained from the soil beneath *Populus trichocarpa* along an ~280 mile north–south corridor in the Pacific Northwest, United States. Additionally, they performed global phylogenetic analysis by incorporating 789 isolates with publicly accessible data from the United States, Japan, and Europe. This analysis identified 34 strongly supported clades using ML and Bayesian methods, with some clades exhibiting intra-and intercontinental distributions. These findings strongly indicate divergence within multiple cryptic species.

Furthermore, the genetic diversity and structure of *C. geophilum* populations were analyzed based on the rDNA ITS2 sequences of 219 ECM root samples collected from 3 plant families (Betulaceae, Fagaceae, and Pinaceae) from 10 forest sites across China (Guo et al., 2021). Analysis of molecular variance (AMOVA) confirmed that genetic differentiation was evident within each geographical population and the population in each host plant family. The Fagaceae population was distinct from the Betulaceae and Pinaceae populations, and the haplotype composition was conspicuously different among the three plant families. These cryptic species may represent locally adapted forms of *C. geophilum*, which have evolved in response to specific environmental conditions. The genetic diversity of angiosperm-associated *C. geophilum* populations is higher than that of gymnosperm-associated populations, suggesting that angiosperm and gymnosperm hosts exert different selective pressures on their symbionts (Vélez et al., 2021). Tedersoo et al. (2024) also provided evidence for niche differentiation in tens of cryptic species of *Cenococcum*, many of which exhibit a preference toward particular partner plant genera.

Currently, it remains uncertain whether *C. geophilum* constitutes a single, highly diverse global species or whether it comprises numerous cryptic species. Subsequent studies could shed light on these local and worldwide relationships by comparing nuclear and mitochondrial genomes from a wide range of geographical isolates along with population genomics approaches.

## 10 Genomics, transcriptomics, and population genomics

### 10.1 Genomics

Within the framework of the Mycorrhizal Genome Initiative (Martin et al., 2011), the nuclear genome of *C. geophilum* (strain 1.58) has been sequenced and annotated by the U.S. Department of Energy Joint Genome Institute (Peter et al., 2016). This genome is the largest among the ECM fungi, with a mapped size of 178 Mbp and a total estimated size of 203 Mbp (Peter et al., 2016; Talhinhas et al., 2017). It is estimated to contain approximately 15,000 genes. In contrast, the genomes of the taxonomically related saprotrophic species *Glonium stellatum* and *Lepidopterella palustris* are approximately four times smaller than that of *C. geophilum*, at 41 and 46 Mbp, respectively, yet they possess similar gene counts to 14,362 and 13,870 predicted gene models, respectively. Phylogenomic analysis using single-copy conserved orthologs confirmed that *C. geophilum* belongs to the class Dothideomycetes, specifically in the order Mytilinidiales, and shares a close evolutionary relationship with the saprotrophic species *G. stellatum and L. palustris*. Despite its close taxonomic relationship with these saprotrophs, *C. geophilum* exhibits unique genomic features consistent with its ECM lifestyle. This is in agreement with the independent origin of ECM ability in *Cenococcum* within the class of otherwise saprobic Ascomycota (Dothideomycetes), with evidence that the most closely related sister group, *Glonium*, is likely saprobic and lacks mycorrhization ability. In their study, Obase et al. (2017) reported that *Pseudocenococcum floridanum* is a more closely related but distinct sister group to other *Cenococcum* lineages and that this new species likely lacks the ability to form ectomycorrhizas. Ongoing sequencing of several strains of *P. floridanum* at JGI^1^ will provide new insights into the evolution of the saprotrophy-to-symbiosis transition in *Cenococcum* clades.

The *C. geophilum* gene repertoire contains 2,176 species-specific genes, including effector-like small secreted proteins (SSPs). Many of these unique genes are involved in protein–protein interactions and signaling mechanisms, which are likely crucial for their symbiotic relationships with plants. Compared with its close relatives, the expanded genome of *C. geophilum* is attributed to its high proportion (81%) of repetitive sequences, primarily composed of transposable elements (TEs). Increased TE content is observed in numerous plant pathogenic fungi, particularly in those with (hemi-) biotrophic lifestyles. This trend is even more pronounced in symbiotic mycorrhizal fungi (Miyauchi et al., 2020; Lebreton et al., 2021). The majority of expanded gene families are associated with TEs or are involved in protein–protein interactions. These families exhibit domains typically observed in proteins related to self/non-self-recognition, which are associated with somatic incompatibility and defense mechanisms, such as HET, NACHT, and WD40 proteins. Notably, the expression of the majority of these gene families remains unregulated in functional mycorrhizas (Peter et al., 2016).

**Figure 4:**
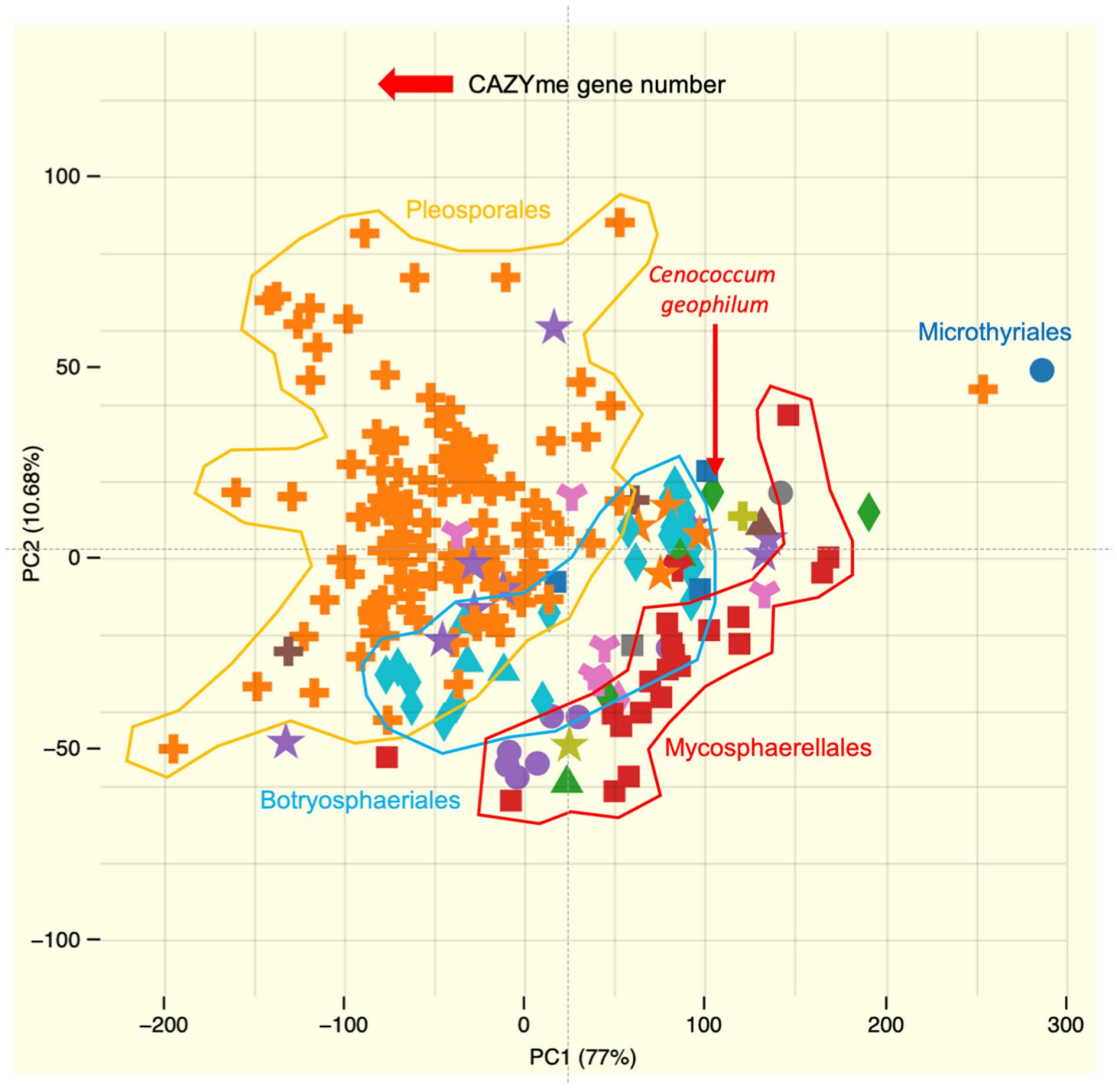
Principal component analysis (PCA) showing the distribution of the CAZyme repertoire in *Cenococcum geophilum* (red arrow) and other sequenced Dothideomycetes available in the JGI MycoCosm database (Grigoriev et al., 2014). Major orders of Dothideomycetes, such as *Botryosphaeriales*, *Mycosphaerellales*, and *Pleosporales*, are also indicated. These data were obtained after semi-manual curation of protein-filtered model sequences by the CAZy team (www.cazy.org) (Drula et al., 2022), and PCA was generated by MycoCosm.

The repertoire of genes encoding Plant cell wall degrading enzymes (PCWDEs) is lower than that of the majority of saprotrophic and pathogenic Dothideomycetes but similar to that of saprotrophic and pathogenic Mycosphaerellales and Botryosphaeriales (Figure 4; Peter et al., 2016). This reduction is striking for enzymes that act on cellulose, hemicellulose, and pectin. Enzymes that act on hemicellulose, such as xylanases (GH10 and GH11), mannanases (GH26), glucuronidases (GH115), and pectin-attacking enzymes (PL1, PL3, PL4, and CE12), are also reduced from two to five members to either none or only one member. Among the sequenced ECM fungi, *C. geophilum* exhibited the most extensive PCWDE repertoire (43 enzymes). Notably, proteins that target crystalline cellulose (GH6, GH7, AA9, and CBM1) are found in the *C. geophilum* genome (Peter et al., 2016) but are frequently absent in other ECM fungi (Kohler et al., 2015; Miyauchi et al., 2020; Lebreton et al., 2021).

**Figure 5:**
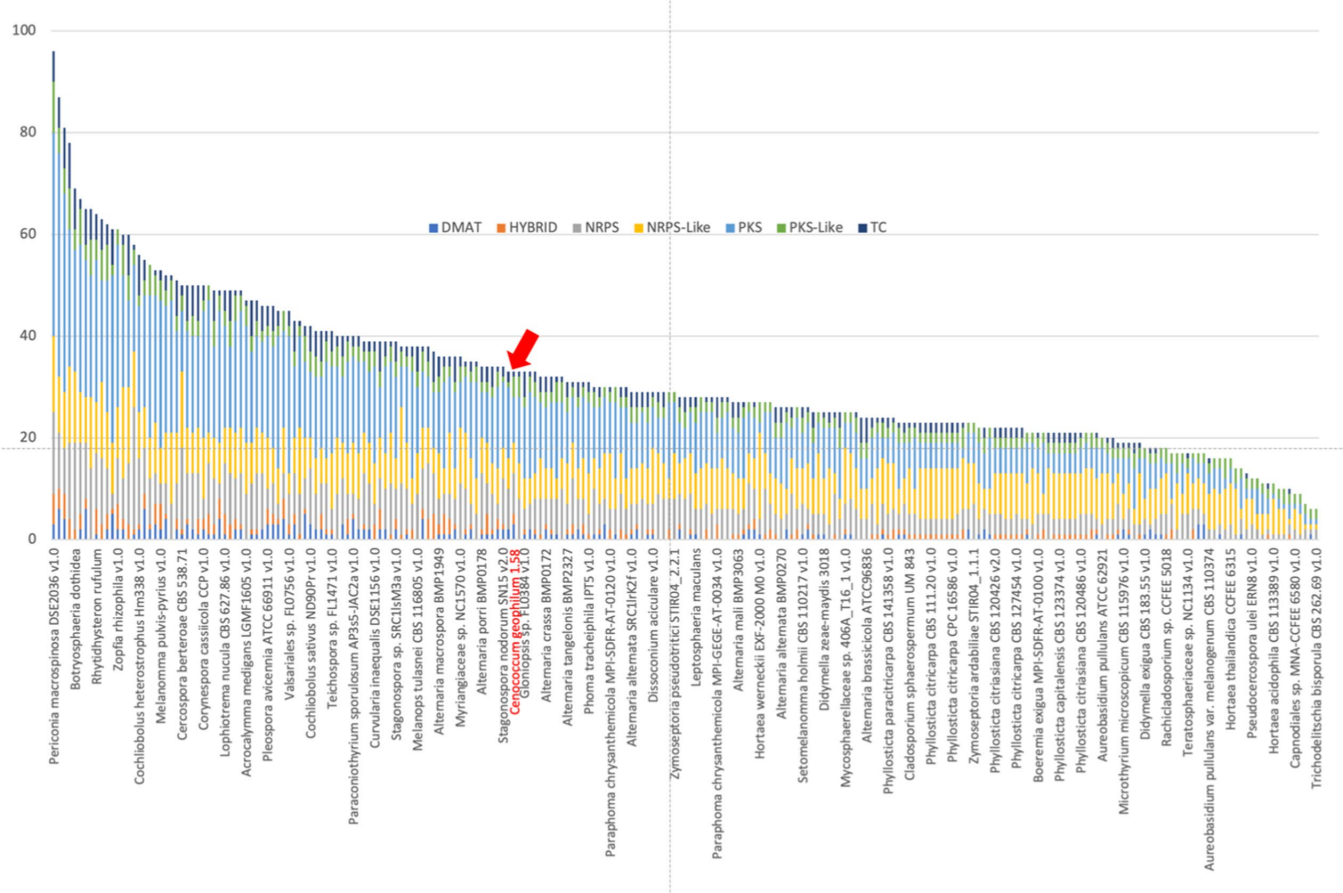
Number of genes coding for secondary metabolism in *Cenococcum geophilum* (red arrow) and other sequenced Dothideomycetes available from the JGI MycoCosm database (Grigoriev et al., 2014). DMAT, prenyltransferase; NRPS, nonribosomal peptide synthase; PKS, polyketide synthase; and TC, terpene cyclase.

With the exception of polyketide synthases (PKSs), the *C. geophilum* genome did not show a reduction in the number of genes associated with the biosynthesis of secondary metabolites (Figure 5; Peter et al., 2016), many of which act as antibiotics in pathogenic interactions and microbial competition in the rhizosphere. These PKSs are typically more numerous than those found in ECM basidiomycetes (Lebreton et al., 2021). In ECM root tips, the expression of the majority of secondary metabolism-related genes is suppressed, except for two non-ribosomal peptide synthases (NRPS) and a PKS (Peter et al., 2016). Notably, one of these NRPS exhibits high protein sequence similarity (42%) to *Aspergillus fumigatus* Pes1, which is involved in the defense against oxidative stress (Reeves et al., 2006). Oxidative stress is an unavoidable consequence of drought and is employed by plants as a defense mechanism against biotic stressors.

### 10.2 Transcriptomics

Peter et al. (2016) revealed that 3% of *C. geophilum* genes were upregulated during symbiosis, as determined by comparing RNA sequences from mycorrhizal roots and free-living mycelia. The most highly expressed and upregulated genes in symbiosis include aquaporins, major facilitator superfamily (MFS) membrane transporters, and small secreted proteins (SSPs), which are proteins less than 300 amino acids in length with a predicted signal peptide (Table 1). Notably, 18–23% of the upregulated genes were specific to *C. geophilum*, with SSPs being overrepresented in these taxon-specific orphan genes compared to their proportion in the overall gene repertoire. These SSPs may function as novel symbiosis-related effectors, similar to the mycorrhiza-induced small secreted proteins (MiSSPs) in *Laccaria bicolor*, which regulates defense-related pathways in host roots (Martin et al., 2016).

**Table 1:** Most highly upregulated genes in ectomycorrhizal roots (ECM) of *Cenococcum geophilum/Pinus sylvestris* compared to free-living mycelia (FLM) (adapted from Peter et al., 2016).

RPKM FLM^1^	RPKM ECM^1^	Fold Change	Protein ID	Definition
0.6	3,849	6,133	690706	Aquaporin (major intrinsic protein)
0.4	550	1,453	600722	Sugar transporter
5.3	5,666	1,073	647346	Aquaporin (major intrinsic protein)
0.1	112	1,018	697512	Unknown function
1.0	476	484	654895	Peptidase
3.7	1,560	420	655146	Unknown function
0.5	185	410	730139	MFS transporter
0.3	91	344	612206	MFS transporter
0.2	51	341	333290	Unknown function
0.3	97	313	600293	Unknown function
2.1	636	297	698167	SSP/unknown function
0.1	36	279	616643	Unknown function
0.1	15	270	660401	SSP/unknown function
0.1	21	236	680403	SSP/unknown function
8.9	2016	227	605087	MFS transporter
0.2	39	225	610797	Amino acid permease
0.9	204	222	608762	Glucose/ribitol dehydrogenase
1.6	363	222	613185	G-protein
2.0	409	207	676136	Ricin B lectin
1.6	325	207	649427	Unknown function

Abbreviations: MFS, major facilitator superfamily; SSP, small secreted proteins. RPKM and fold change values are represented using a gradient from white to dark red, with the intensity increasing in line with the values.^1^Mean of three replicates; given in reads per kilobase of transcript per million reads mapped (RPKM).

De Freitas Pereira et al. (2018) compared the secretome of *C. geophilum* interacting with pine and poplar trees; they reported that the levels of transcripts encoding carbohydrate-active enzymes (CAZymes) and MiSSPs were strikingly different. This may be related to the different cell wall compositions of the pine and poplar roots (Sarkar et al., 2009). Colonizing *C. geophilum* hyphae may require different cell wall-loosening enzymes to penetrate the roots and effectors to dampen the host immune system.

The gene expression analysis revealed significant changes in the expressions of two aquaporins (AQP) that encode water channels during symbiosis in the ECM rootlets of *Pinus sylvestris* (Table 1; Peter et al., 2016). The substantial increase in water-permeable AQPs in symbiotic rootlets may be triggered by the water and/or nutrient requirements of the plant during interactions. Studies on expression have shown downregulation and upregulation of these AQP genes under drought conditions (a shoot water potential of −3.5 MPa). Intriguingly, under well-watered control conditions, the transcript levels of the drought-induced classical AQP (Cenge3:604158) correlated best with the shoot water potential of their host plant. In the same drought/re-watering experiment, Peter et al. (2016) evaluated the conditions of mycorrhizal plants compared with non-mycorrhizal plants. They noted significantly higher needle N content, net photosynthesis, and water use efficiency in ECM pine seedlings than those in their non-mycorrhizal counterparts, confirming the mutually beneficial relationship between fungi and plants. However, mycorrhizal inoculation had no significant effect on drought treatment.

Zhang et al. (2024) investigated the effects of several *C. geophilum* ecotypes on the drought resistance of *Pinus massoniana* seedlings. They found that inoculation with various strains of *C. geophilum* improved the drought resistance of seedlings by affecting the water content, photosynthesis, osmotic adjustment substances, and antioxidant enzyme activities. The transcriptomic analysis revealed that the seedlings primarily regulate their energy metabolism and redox reactions to cope with early drought stress. The effectiveness of inoculation did not depend on the drought tolerance of the *C. geophilum* ecotype; that is, the drought resistance of the mycorrhizal seedlings did not correlate with the inherent drought resistance of the *C. geophilum* strain itself. The beneficial effects of *C. geophilum* inoculation on the growth of pine seedlings during the early stages of drought stress suggest that this symbiont can be used in reforestation programs in drought areas. Using 1-D gel electrophoresis and LC–MS/MS, Kerner et al. (2012) identified 12 proteins that were differentially accumulated in mycelia subjected to drought conditions compared to controls. The induced responses in *C. geophilum* point toward the regulation of osmotic stress, maintenance of cell integrity, and counteracting increased levels of reactive oxygen species formed during water deprivation.

The survival of *C. geophilum* in various environments depends on its ability to regulate stress-related gene expression. Transcriptome profiling has shown that *C. geophilum* can enhance the expression of numerous genes associated with stress resistance, including those associated with osmotic/drought stress (Li et al., 2023), salt stress (Li et al., 2022), oxidative stress, heat shock responses (Yan et al., 2022), and heavy metal tolerance (Shi et al., 2022). These genes, which are involved in processes such as organic acid secretion, antioxidant activity (e.g., peroxidase, superoxide dismutase, and ubiquinone), membrane transport, and sphingolipid metabolism pathways, are regulated in a coordinated manner. This suggests that their expression is controlled by transcription factors that react to environmental changes, such as heat shock factors and elements responsive to osmotic stress. Verification of the functional roles of the numerous identified stress-related genes will necessitate genetic transformation protocols to inactivate them through RNA interference silencing or CRISPR/Cas9.

Although identifying differentially expressed genes in mycelia cultivated under laboratory conditions represents a promising approach to characterizing genes involved in drought stress adaptation, it is important to consider that gene expression in natural environments may differ significantly, as demonstrated in a recent study by Pellitier et al. (2024). They investigated fungal communities inhabiting the roots of *Populus trichocarpa* distributed across a precipitation gradient in the Pacific Northwest, United States. These communities were analyzed using taxonomic (metabarcoding) and functional (metagenomic) approaches. Their findings revealed that fungal genes associated with drought stress tolerance and plant water uptake (including genes for melanin synthesis, hydrophobins, aquaporins, trehalose synthases, and other gene families) were not predominant in drier soils.

### 10.3 Population genomics

Dauphin et al. (2021) conducted a study on 16 European isolates of *C. geophilum* using whole-genome resequencing. Their findings revealed divergent lineages in geographically confined sampling locations, without strong geographic structuring. Genome-wide polymorphism analyses indicated species subdivisions and suggested two primary genetic groups: clonal and recombinant. The lineage phylogeny and groupings were largely corroborated by the numerous gene copy number variations (CNVs) discovered among the genomes. Although the clonal cluster contained nearly twice as many strains, gene diversity analyses showed a higher genetic diversity in the recombinant group. Based on Tajima’s D statistics, the top candidate genes potentially under positive selection differed between the two groups. The recombinant cluster exhibited more genes from lineage-specific expanded gene families involved in self/non-self-recognition, whereas the clonal cluster showed genes related to secondary metabolism. Additionally, this study confirmed *C. geophilum* heterothallism through chromosomal synteny analysis of the mating genes *MAT1-1* and *MAT1-2* idiomorphs. It also revealed significant genetic rearrangements in the surrounding coding and non-coding regions of the strains carrying both the same and opposite *MAT1* idiomorphs. These results highlight the complex genome architecture of *C. geophilum*, possibly due to cryptic sex-and/or transposon-related mechanisms.

Lian et al. (2024) assembled five *C. geophilum* genomes representing different geographical regions and generated a pan-genome comprising 7,556 core gene families and 12,686 dispensable gene families. Genome resequencing of 304 isolates with worldwide distribution was performed to estimate the genetic diversity, structure, and demographic history of *C. geophilum* isolates. Millions of single nucleotide polymorphisms (SNPs) and 0.04–0.2% structural variations have been identified, suggesting the occurrence of several ecotypes with different drought resilience levels. Their genome-wide association and transcriptome analyses identified 161 genomic regions that were significantly associated with 9 biological and environmental adaptation traits, encompassing 2,738 potential genes, including EVM0002574, which are associated with resistance to cadmium, salt, and high-temperature stresses. These genomic resources and diversity datasets provide valuable tools and a comparative genomic framework for investigating ectomycorrhizal symbiotic relationships.

## 11 Applications in forestry and conservation

*Cenococcum geophilum* is a highly adaptable ECM fungus that demonstrates significant potential for ecological restoration and environmental remediation through microbial engineering. The symbiont forms a dense network of melanized hyphae around the roots of host plants, creating a protective sheath. This symbiotic association is particularly beneficial in water-limited environments, where *C. geophilum* helps the host tree maintain hydraulic conductivity and photosynthetic activity under drought stress. Additionally, the fungus has been shown to enhance salt tolerance of host plants, making it valuable for reclaiming saline soils. Finally, its extensive distribution, broad host range, and high stress tolerance make it particularly valuable for addressing desertification and adapting to climate change (Zhai et al., 2023). Through the utilization of genomics and other-omics techniques, we acquired a more comprehensive understanding of the molecular, physiological, and ecological mechanisms underlying the establishment and functioning of *C. geophilum* ECM under environmental stress. Candidate genes related to adaptation to these environmental stresses can be used to select appropriate strains for the mycorrhizal inoculation of tree seedlings in environments prone to drought or other abiotic stresses. Surveys of soil fungal communities using DNA metabarcoding can be used to predict the environmental conditions under which *C. geophilum* inoculation is beneficial for forest management and restoration. This enhanced knowledge should be leveraged to develop practical applications, such as mycorrhizal inoculation or microbial engineering, which would enhance ecosystem function and preservation, aid in alleviating climate change impacts, and maintain the sustainability of forest ecosystems.

Furthermore, *C. geophilum* colonizes herbaceous plants. By forming associations with both woody and non-woody plants, symbionts can contribute to the development of diverse plant communities in challenging environments. Its ability to support multiple plant species can increase soil stability, reduce erosion, and improve nutrient cycling in degraded ecosystems. In arid regions, *C. geophilum* colonizes both ECM trees and Cistaceae plants. The physical connection of *C. geophilum* mycelial networks with both tree roots and herbaceous plants could redistribute water from the deeper roots of the tree, retain a portion of the water in the upper soil layers, and facilitate enhanced nutrient acquisition by the host plants. Similarly, the mouse-tail bog sedge (*Carex (Kobresia) myosuroides*) can be incorporated into tree plantations in northern and alpine regions. In environments contaminated by industrial waste, *C. geophilum* has shown promising results in the remediation of soils affected by heavy metals and petroleum (Danielson and Visser, 1989). The fungus has also exhibited the capacity to accumulate and sequester various heavy metals, including Pb, Cd, and Zn, in its melanized cell walls (Huang et al., 2014; Azaiez et al., 2018; Shi et al., 2022; Zhang et al., 2023,). This characteristic renders *C. geophilum* a potential candidate for mycoremediation of polluted soils. Moreover, their association with host plants can enhance phytoremediation efforts by improving plant survival and growth at contaminated sites.

Urban environments often present challenging conditions for plant growth such as soil compaction, elevated temperatures, and air pollution. *C. geophilum* is frequently the most abundant ECM symbiont found in the roots of urban trees (Garbaye and Churin, 1996; Hui et al., 2017; Van Geel et al., 2018; Olchowik et al., 2021). Its ability to form symbiotic relationships can improve resilience to these stressors, potentially leading to increased tree longevity and enhanced ecosystem services in urban areas.

## 12 Future research

Several enduring challenges persist in utilizing genomics and other-omics approaches to enhance our understanding of the biology and ecology of *C. geophilum*, including its evolutionary history, developmental processes, functional aspects, and its resilience to environmental stress. We have identified several critical questions that require further investigation:

1. What molecular mechanisms underlie the genetic diversity of *C. geophilum*, and how does this genetic polymorphism facilitate its worldwide distribution?
2. What are the transcriptional regulators and gene networks that drive the resilience of *C. geophilum* to extreme environmental conditions including drought stress, heavy metal contamination, and high salinity?
3. What role do epigenetic modifications play in the ability of *C. geophilum* to adapt to various environments?
4. What is the significance of horizontal gene transfer (if any) in the evolutionary trajectory of *C. geophilum*?
5. How does the mutualistic association between *C. geophilum* and its plant partners fluctuate across various environmental settings?
6. What patterns have emerged in the *C. geophilum* population genomics across different geographical regions? How will climate change alter symbiont distribution worldwide?
7. How do the secondary metabolites produced by *C. geophilum*, such as melanin, influence its interactions with soil microbial communities including soil and litter decomposers?

The role of *C. geophilum* in ecosystem resilience is becoming increasingly important in the context of climate change. As extreme weather events and environmental stressors become more frequent, the capacity of this fungus and other mycorrhizal fungi to support plant growth and survival under adverse conditions may be crucial for maintaining ecosystem stability and biodiversity. Furthermore, their potential to enhance carbon sequestration through increased plant growth and soil organic matter accumulation may contribute to climate-change mitigation.

## Footnotes

^1^ https://mycocosm.jgi.doe.gov/mycocosm/home
